# Rapid Molecular Detection of Rifampicin Resistance Facilitates Early Diagnosis and Treatment of Multi-Drug Resistant Tuberculosis: Case Control Study

**DOI:** 10.1371/journal.pone.0003173

**Published:** 2008-09-09

**Authors:** Philly O'Riordan, Uli Schwab, Sarah Logan, Graham Cooke, Robert J. Wilkinson, Robert N. Davidson, Paul Bassett, Robert Wall, Geoffrey Pasvol, Katie L. Flanagan

**Affiliations:** 1 Department of Infection and Tropical Medicine, Lister Unit, Northwick Park Hospital, Harrow, Middlesex, United Kingdom; 2 Wellcome Centre for Clinical Tropical Medicine, Division of Medicine, Imperial College London, London, United Kingdom; 3 Department of Medical Microbiology, Northwick Park Hospital, Harrow, Middlesex, United Kingdom; University College London, United Kingdom

## Abstract

**Background:**

Multi-drug resistant tuberculosis (MDR-TB) is a major public health concern since diagnosis is often delayed, increasing the risk of spread to the community and health care workers. Treatment is prolonged, and the total cost of treating a single case is high. Diagnosis has traditionally relied upon clinical suspicion, based on risk factors and culture with sensitivity testing, a process that can take weeks or months. Rapid diagnostic molecular techniques have the potential to shorten the time to commencing appropriate therapy, but have not been put to the test under field conditions.

**Methodology/Principal Findings:**

This retrospective case-control study aimed to identify risk factors for MDR-TB, and analyse the impact of testing for rifampicin resistance using RNA polymerase B (*rpoB*) mutations as a surrogate for MDR-TB. Forty two MDR-TB cases and 84 fully sensitive TB controls were matched by date of diagnosis; and factors including demographics, clinical presentation, microbiology findings, management and outcome were analysed using their medical records. Conventionally recognised risk factors for MDR-TB were absent in almost half (43%) of the cases, and 15% of cases were asymptomatic. A significant number of MDR-TB cases were identified in new entrants to the country. Using *rpoB* mutation testing, the time to diagnosis of MDR-TB was dramatically shortened by a median of 6 weeks, allowing patients to be commenced on appropriate therapy a median of 51days earlier than those diagnosed by conventional culture and sensitivity testing.

**Conclusions/Significance:**

MDR-TB is frequently an unexpected finding, may be asymptomatic, and is particularly prevalent among TB infected new entrants to the country. Molecular resistance testing of all acid fast bacilli positive specimens has the potential to rapidly identify MDR-TB patients and commence them on appropriate therapy significantly earlier than by conventional methods.

## Introduction

There has been a resurgence of tuberculosis (TB) in the United Kingdom (UK) since 1987, with an overall annual incidence in England and Wales of 14 per 100, 000 in 2005 [1]. In London, the incidence reached 46 per 100,000 in 2005, accounting for 45% of all TB cases notified in the UK that year [1]. Multi-drug resistant tuberculosis (MDR-TB) (by definition TB caused by *Mycobacterium tuberculosis* resistant to at least rifampicin and isoniazid) requires prolonged therapy with a combination of second line anti-tuberculous drugs, many of which are less effective, more toxic and more expensive than first line drugs [2]. Cases of suspected or confirmed MDR-TB may require prolonged inpatient management, extended periods of outpatient follow-up and present a significant financial burden. In the UK the proportion of MDR-TB in initial isolates has remained stable at 0.9–1.9% between 1994 and 2005 [1], [3], [4]. However, MDR-TB cases are not uniformly distributed geographically, with higher than average frequencies occurring in certain areas and population groups, particularly in London and amongst asylum seekers [5].

A key element in the management of MDR-TB is the early institution of an appropriate treatment regimen. Traditionally, diagnosis has relied upon culture and sensitivity testing, a process which can take weeks and sometimes months. Such a delay results in increased patient morbidity and mortality, and may also lead to the spread of MDR-TB both within the community and to healthcare workers [6]. The development of rapid diagnostic techniques for multi-drug resistance testing in tuberculosis cases is likely to provide a solution to this problem. A rapid molecular test analysing for a mutation in the 69 base pair region (codon 507–533) of the gene encoding the beta chain of RNA polymerase (*rpoB*) arises in 95% of rifampicin resistant *M. tuberculosis* strains [7]. Since 83% of rifampicin resistant strains in the UK are also isoniazid resistant, rifampicin resistance may be considered a surrogate marker for MDR-TB [8], [9]. Since 1997 all acid fast bacilli (AFB) positive samples in our hospital have routinely been sent to the Mycobacterium Reference Unit for testing for *rpoB* mutations using the INNO-LiPA Rif.TB assay (Immunogenetics, Zwijndrecht, Belgium) [9], [10]. We undertook this study to examine risk factors for MDR-TB in our patient population, to assess the impact of the introduction of *rpoB* testing on clinical practice, and to determine whether this test would hasten MDR-TB diagnosis in patients where recognised risk factors did not apply, and thereby translate into tangible benefits for both patients and their contacts.

## Methods

Wherever appropriate we used the Strengthening the Reporting of Observational Studies in Epidemiology (STROBE) recommendations to improve the quality of our study [11], [12].

### Cases and controls

Northwick Park Hospital (NPH) is a university-affiliated district general hospital in Northwest London, UK. Patients with suspected TB come from three main sources; the local population, the Health Control Units (HCUs) at London's Heathrow and Gatwick airports, and via tertiary referrals from other hospitals. The HCUs screen new entrants (selected new immigrants, long-stay visitors (>6 months), students from high risk countries and all asylum seekers) with a chest x-ray (CXR) on arrival in the UK. Those with a CXR suspicious of TB are generally referred to NPH for assessment.

We retrospectively reviewed all cases of culture proven MDR-TB diagnosed over a 22 year period between January 1982 and December 2004 at NPH. Cases were identified by searching microbiology laboratory records, inpatient diagnostic databases and TB notifications for the local districts of Brent & Harrow. Two patients found to be HIV positive were excluded from the study. A control group of patients with fully sensitive TB (*M. tuberculosis* sensitive to rifampicin, isoniazid , pyrazinamide , ethambutol and streptomycin) was obtained by selecting the preceding and subsequent fully sensitive cases for each MDR-TB patient. Where the medical records of these control cases were unavailable we used the next available appropriate case. Thus, for each MDR-TB case we obtained two controls. By using this method we were able to control for changes in presentation and management that might have occurred over the time span of this study.

### Analysis of case notes

For each case we assessed the patient's demographics, clinical presentation, microbiology findings, management and outcome. The date of diagnosis of MDR-TB was defined as the date of recognition, confirmed either by a positive probe for an *rpoB* mutation or culture sensitivity results confirming at least rifampicin and isoniazid resistance. Cure was defined as clinical with or without confirmed microbiological resolution of the disease without relapse of symptoms or positive culture. Cases with smear positive pulmonary TB were confined in hospital until 3 successive sputum specimens were found to be AFB smear negative. If these 3 specimens were subsequently found to be culture negative then no further specimens were taken unless clinically indicated i.e. a worsening chest X-ray, ongoing fevers, continued sputum production. If any of the 3 specimens were culture positive then samples were collected in clinic until two or more consecutive samples were TB culture negative. In many cases symptoms resolved on treatment and thus there was no possibility of obtaining repeat specimens i.e. no longer producing sputum, CXR cleared, resolved lymphadenopathy. Treatment duration was the time on appropriate anti-tuberculous therapy, and the time of post treatment follow-up was defined as the interval between cessation of therapy and the last known contact with the patient. An adverse drug reaction was defined as symptoms that resulted in the suspension or cessation of one or more drugs. A commercial radioimmunoassay was used to measure 25 hydroxy-vitamin D levels (DiaSorin Ltd, Wokingham, Berks, UK).

### Statistical Methods

Analysis of factors influencing whether patients had MDR-TB or not were measured on a categorical scale and the effect of each variable was examined using logistic regression. The individual effect of each variable was first examined separately using univariate analysis, and then the combined effect of the explanatory variables on the occurrence of MDR-TB was examined in a multivariate analysis. Only those factors that showed evidence of a significant effect in the univariate analysis were included in the multivariate analysis. A backwards selection procedure was used to determine the final model, which involved removing all non-significant variables one at a time until all remaining variables were statistically significant.

The paired t-test was used to compare the time to diagnosis using standard TB cultures with the time to diagnosis by *rpoB* mutation testing for those patients who had values for both. The outcomes for MDR-TB cases and controls were compared using Fisher's exact test for categorical outcomes. For continuous outcomes the 2 groups were compared using either the unpaired t-test for normally distributed factors, or the Mann-Whitney U test for non-normally distributed outcomes.

## Results

**Figure 1:**
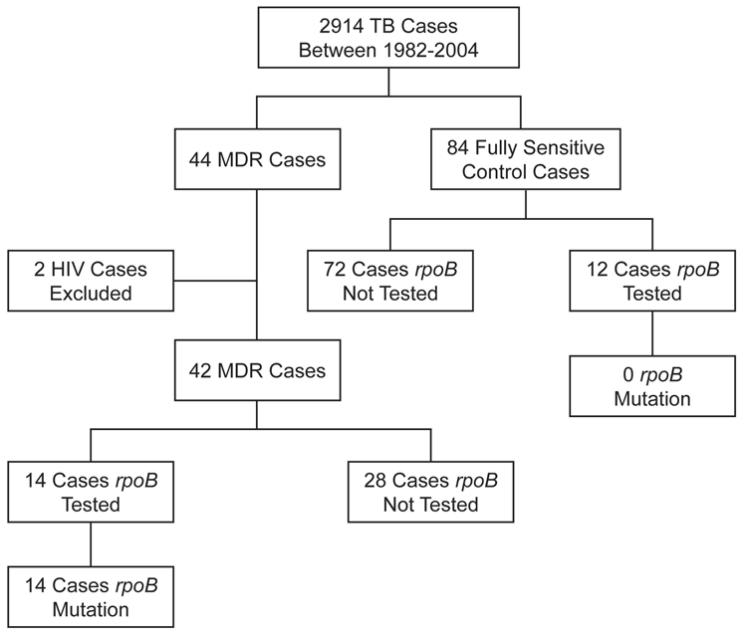
Flow chart illustrating the 22 year case-control patients selected for the study.

Between 1982 and 2004, of 2,914 TB patients managed at NPH, 44 (1.5%) were found to have MDR-TB (Figure 1). Two patients found to be HIV positive were excluded, leaving a total of 42 patients in our study. Data was collected for 84 fully sensitive control cases. Controls were found to be representative of the entire TB cohort in terms of geographical region of origin and source of referral (data not shown).

The majority of MDR-TB cases (24/42, 57%) were referred from the airports, whereas non MDR-TB cases (63/84, 75%) were more often from the local resident population (p<0.001) (Table 1). Neither age nor sex influenced the likelihood of having MDR-TB (p = 0.55 and 0.9 respectively) (Table 1). The non-MDR cases had been in the UK significantly longer than MDR-TB cases (median 32 months and 0 months respectively, p = 0.004), but this was no longer significant after multivariate analysis (Table 1). Patients from West Africa were overrepresented in the MDR-TB group; 73% had MDR-TB, constituting 19% of all MDR-TB cases in this series (Table 1). Forty four percent of patients from South East Asia (SEA) and 38% of those from the Indian subcontinent (ISC) had MDR-TB and the lowest occurrence was in those of European/Middle Eastern origin (24%). The most frequent ethnic group among the controls were those from the ISC (42 cases), who constituted 50% of fully-sensitive TB cases. Despite these differences, the numbers in each group were small and country of origin was not a significant risk factor in univariate analysis (p = 0.08) (Table 1).

**Table 1:** Comparison of risk factors amongst MDR-TB cases and controls.

Variable	Group	MDR-TB N (%)	Controls N (%)	Odds Ratio (95% CI)	P-value univariate	P-value multivariate
Age *	NA	NA	NA	0.93 (0.74, 1.17)	0.55	NS
Sex	Female	21 (34%)	41 (66%)	1		
	Male	21 (33%)	43 (67%)	0.95 (0.45, 2.00)	0.9	NS
Geographical regions of origin	SEA	4 (44%)	5 (56%)	1		
	ISC	16 (38%)	42 (62%)	0.48 (0.11, 2.00)		
	West Africa	8 (73%)	3 (27%)	3.33 (0.51, 21.6)		
	Africa - other	9 (31%)	20 (69%)	0.56 (0.122, 2.6)		
	Europe/Middle East	4 (24%)	13 (76%)	0.38 (0.07 ,2.17)	0.08	NS
	Unknown	1	1			
Referral source	Airport	24 (57%)	18 (43%)	1		
	Local	13 (17%)	63 (83%)	0.15 (0.06, 0.36)		
	Tertiary	4 (57%)	3 (43%)	1.00 (0.20, 5.04)	**<0.001**	**0.003**
	Unknown	1	0			
Time in UK	0–1 months	25 (58%)	18 (42%)	1		
	2–12 months	2 (22%)	7 (78%)	0.21 (0.04, 1.11)		
	13–60 months	5 (18%)	23 (81%)	0.17 (0.05, 0.54)		
	>60 months	8 (26%)	23 (74%)	0.25 (0.09, 0.69)	**0.004**	NS
	Unknown	2	13			
TB contact	No	38 (36%)	69 (64%)	1		
	Yes	3 (18%)	14 (82%)	0.39 (0.11, 1.44)	0.16	NS
	Unknown	1	1			
Previous TB diagnosis	No	15 (16%)	79 (84%)	1		
	Yes	24 (83%)	5 (17%)	23.7 (7.9, 71.4)	**<0.001**	**<0.001**
	Unknown	3	0			

* Odds ratio given for a 10 year increase in age.

NA = Not applicable.

NS = Not significant.

SEA = Southeast Asia.

ISC = Indian subcontinent (India, Pakistan and Sri Lanka).

Previous TB was a strong predictor for MDR-TB (p<0.001), with 57% of MDR-TB patients having previously had TB compared to only 6% of the controls (Table 1). A history of previous contact with a known TB case was not predictive (p = 0.16) (Table 1). Of significance, in 43% of the MDR-TB cases there were no identifiable risk factors at all for the presence of MDR-TB. Furthermore, there were no clinical features that distinguished MDR-TB from sensitive TB cases. Both groups were equally likely to be asymptomatic at presentation (p = 0.19), to have a cough (p = 0.24), fever (p = 0.33), weight loss (p = 1.0), or lymphadenopathy (p = 1.0) (data not shown). The proportion of specimens containing acid fast bacilli (from sputum, gastric washings, bronchoalveolar lavage or fine needle aspirate of a lymph node) was comparable in the MDR-TB and control groups (p = 0.11–1.0) (Table 2).

**Table 2:** Comparison of methods of diagnosis in MDR-TB cases and controls.

Variable	Group	MDR-TB N (%)	Controls N (%)	P-value
Sputum AFB	Negative	9 (29%)	12 (38%)	0.6
	Positive	22 (71%)	20 (62%)	
GW AFB	Negative	12 (55%)	13 (57%)	1
	Positive	10 (45%)	10 (43%)	
BAL AFB	Negative	5 (45%)	14 (78%)	0.11
	Positive	6 (55%)	4 (22%)	
FNA AFB	Negative	5 (100%)	16 (73%)	0.56
	Positive	0 (0%)	6 (27%)	
Asymptomatic	No	33 (85%)	78 (93%)	0.19
	Yes	6 (15%)	6 (7%)	
*RpoB* mutation	No	0 (0%)	12 (100%)	**<0.001**
	Yes	14 (100%)	0 (0%)	
Time to *rpoB* diagnosis	NA	9 (8, 26)*	8 (6, 10)*	0.11
Time to culture sensitivity diagnosis	NA	51 (42, 71)*	56 (46, 79)*	0.1

* Median (inter-quartile range (IQR)).

AFB = acid fast bacilli, GW = gastric washing, BAL = bronchoalveolar lavage.

FNA = fine needle aspiration of a lymph node/TB mass.

NA = Not applicable.

Since 1997 all samples found to contain acid fast bacilli were routinely tested for the *rpoB* mutation. Since then, 100% (14/14) of those patients subsequently confirmed to have MDR-TB tested positive for the *rpoB* mutation compared to none (0/12) of the control patients (p<0.001) (Figure 1). One patient tested positive for an *rpoB* mutation, but the organism was subsequently found to be rifampicin mono-resistant on culture and was therefore excluded from this study. The median time taken to obtain the *rpoB* result was 9 days (range 7–43) in the MDR-TB group and 8 days (range 4–55) in the control group (Table 2). In comparison, the time to obtain sensitivity results through standard mycobacterial culture and sensitivity testing was 51 days (range 33–115) in the MDR-TB group, and 56 days (range 33–119) in the control group. Thus *rpoB* testing provided a result 6–7 weeks earlier in both groups (42 days and 48 days respectively) compared to conventional culture and sensitivity testing (p<0.001). Of the 28 MDR-TB cases that did not have an *rpoB* test for any reason, 17 (61%) were positive for AFB on microscopy, and therefore could potentially have been diagnosed and correctly treated earlier if the *rpoB* test was used.

Other investigations were unhelpful in distinguishing MDR from sensitive TB cases. The proportion of patients with a positive tuberculin skin test (TST) was comparable between the 2 groups for Mantoux (p = 1.0) and Heaf (p = 1.0) tests (data not shown), and full blood count, urea and electrolytes and liver function tests were also comparable. The MDR-TB patients had significantly lower CRP values (MDR-TB median 11 mg/L, control group median 32 mg/L, p = 0.03), significantly higher lymphocyte counts (MDR-TB mean 1.8×109/L, control group mean 1.5×109/L, p = 0.04), and significantly more MDR-TB patients had normal vitamin D levels (MDR-TB 54%, control group 12%, p = 0.007) (data not shown), although none of these factors could be used to distinguish MDR-TB from a fully sensitive case.

The length of inpatient hospital stay was significantly longer in the MDR-TB group, with a median of 32 days for MDR-TB patients and 9 days for controls (p<0.001) (data not shown). As expected, the duration of treatment was much longer in the MDR-TB group with a median total duration of treatment of 18 months (inter-quartile range (IQR) 13–24) in the MDR-TB group, compared to 6 months (IQR 6–9) in the control group (p<0.001) (Table 3). The MDR-TB patients suffered significantly more adverse effects than those in the control group (47% of MDR-TB patients, 21% of controls, p = 0.003) (data not shown). There was also an extended period of follow-up post treatment for MDR cases compared to controls, with a median of 11 months (IQR 1–24) in the MDR-TB group and 3 months (IQR 1–9) in the control group (p = 0.05) (Table 3). The outcomes differed significantly between the two groups (p = 0.03) (Table 3). Overall, among those for whom outcome data was available, all the control group and 86% of MDR-TB cases were cured. There were no known deaths or treatment failures in the control group. For the 22 sputum positive MDR-TB patients the time between treatment start and culture negativity was a median of 99 days, mean of 163 days. Only one of the control group had a repeat sputum cultured as an outpatient, and this was found to be culture negative. In the MDR-TB group the treatment failed in one case (2%) and three patients died (7%). Outcome data was not available in 25% of controls and 31% of MDR-TB cases due to patients being lost to follow-up, transferred to another hospital or returning to their country for origin (Table 3). Surgical intervention was required for 6 (14%) of the MDR-TB cases and 3 (4%) of the control group (p = 0.06) (Table 3).

**Table 3:** Comparison of outcomes for MDR-TB cases and controls.

Variable	Group	MDR-TB N (%)	Controls N (%)	P-value
Surgical intervention	No	35 (86%)	79 (96%)	0.06
	Yes	6 (14%)	3 (4%)	
Treatment duration (months)*	NA	18 (13, 24)	6 (6, 9)	**<0.001**
Follow-up post treatment (months)*	NA	11 (1, 24)	3 (1, 9)	**0.05**
Outcome	Cure	25 (60%)	63 (75%)	**0.03**
	Failed/relapsed	1 (2%)	0 (0%)	
	Died	3 (7%)	0(0%)	
	Lost to follow-up	4 (10%)	4 (5%)	
	Follow-up elsewhere	4 (10%)	12 (14%)	
	Return to C of O	5 (12%)	5 (6%)	

* Median (interquartile (IQR) range).

C of O = country of origin.

NA = not applicable.

Median treatment times and outcomes were comparable for MDR-TB patients regardless of whether the diagnosis was made by *rpoB* mutation or standard culture (p = 0.35 and 0.9 respectively) (Table 4). A significant proportion of those MDR-TB cases diagnosed by culture never received the correct treatment (36% of culture diagnosed, 0% of *rpoB* diagnosed cases, p = 0.02) (Table 4). The reasons for this include patient death before treatment was initiated, moving away before treatment was started or not receiving the correct treatment for their sensitivities. Most of the cases where the patients did not receive the correct treatment for their sensitivities occurred early on in the case series. Furthermore, those diagnosed by the presence of an *rpoB* mutation were followed up for less time (median 7 months vs. 13 months), although this difference was not significant in our case series (p = 0.32) (Table 4). Five of the 6 MDR-TB patients requiring surgery were diagnosed by culture and sensitivity rather than by *rpoB* test. This raises the possibility that delay in diagnosis resulted in disease progression and the need for surgical intervention, although the numbers were too small to be significant (p = 0.65) (Table 4). None of the MDR-TB patients diagnosed by *rpoB* probe died, whereas 11% (3/28) of those MDR-TB cases diagnosed by culture died (Table 4), although again numbers were too small to test for significance.

**Table 4:** Comparison of MDR-TB cases diagnosed by *rpoB* mutation versus culture alone.

Variable	*RpoB* diagnosed MDR-TB	Culture diagnosed MDR-TB	P-value
Number of patients	14	28	
Median (IQR) in-patient days	51 (16, 81)	23 (9, 78)	0.56
**Diagnosis and Treatment**			
Median (IQR) time to *rpoB* diagnosis (days)	9 (8, 26)	NA	
Median (IQR) time to culture diagnosis (days)	50 (38, 71)	52 (44, 76)	0.32
Median (IQR) time to starting correct treatment (days)	8 (1, 18)	59 (25, 95)	**0.008**
Cases (%) where MDR-TB treatment was not started for any reason	0 (0%)	10 (36%)	**0.02**
*Cases (%) where correct treatment never started*	*0 (0%)*	*5 (18%)*	*0.15*
*Cases (%) died before correct treatment could be instituted*	*0 (0%)*	*3 (11%)*	*0.54*
*Cases (%) followed up elsewhere before MDR diagnosis*	*0 (0%)*	*2 (7%)*	*0.55*
Cases (%) that underwent surgery	1/14 (7%)	5/28 (18%)	0.65
Median (IQR) length of treatment (months)	19 (18, 24), (n = 10)	18 (13, 24), (n = 23)	0.35
Median (IQR) length of follow-up (months)	7 (0, 17), (n = 11)	13 (1, 40), (n = 16)	0.32
Cases (%) that experienced any AE of drug treatment	7/14 (50%)	12/26 (46%)	1.00
**Outcome**			
Cure	10 (71%)	15 (54%)	0.9
Failure/relapse	0 (0%)	1 (4%)	
Lost to follow-up	1 (7%)	3 (11%)	
Transferred to another institution	1 (7%)	3 (11%)	
Death	0 (0%)	3 (11%)	
Return to Country of Origin	2 (14%)	3 (11%)	

IQR = interquartile range.

AE = adverse event.

## Discussion

MDR-TB poses major management, public health and diagnostic problems, and is often not considered until culture and first line sensitivity tests become available, which may be 8 weeks or more after presentation. Indeed, in our series 43% of the MDR-TB cases had no identifiable risk factors and were thus unexpected. MDR-TB patients are often treated with conventional quadruple therapy in the first instance, with the risk of development of further resistance to pyrazinamide and/or ethambutol whilst awaiting sensitivities. Advances in molecular diagnostic techniques have the potential to significantly hasten the diagnosis and institution of adequate therapy. Our retrospective case-control study of all non-HIV-related cases of MDR-TB diagnosed at our hospital over a 22 year period has shown, for the first time, the significant impact that rapid molecular testing for rifampicin resistance can make on the diagnosis and management of MDR-TB in clinical practice.

The introduction of routine *rpoB* gene testing for all AFB positive samples improved the time to diagnosis of MDR-TB by 6–7 weeks compared to relying on culture and sensitivity testing. Furthermore, a ‘negative’ *rpoB* test provided reassurance that the organism was likely to be fully sensitive significantly earlier than culture testing. As a result, MDR-TB patients diagnosed by *rpoB* probe were commenced on appropriate MDR-TB treatment regimens at least 7 weeks earlier than those for whom standard culture and sensitivity testing was relied upon. This could be critical in preventing disease progression, further drug resistance and spread to other individuals.

The only other factor that was consistently predictive of MDR-TB in our series was a previous history of TB, which was present in 57% of cases. This confirms the findings of others [13]–[15] and applied regardless of whether the patient received their treatment in the UK or abroad. Surveillance data on MDR-TB prevalence throughout the world remains sparse [16], [17], but our results suggest that the region (or country) of origin cannot be used to rule out MDR-TB. In our series MDR-TB cases were more likely to have been referred from the HCUs at airports than from the local community. HCU referral is at the discretion of the immigration officer and is based upon risk of TB in the country of origin (>40 per 100, 000), as well as the intended length of stay (usually >6 months). UK studies indicate that approximately 60% of new entrants with active TB are not identified until they later present to hospital [18], [19]. This is likely to include cases of MDR-TB entering the community, with the potential for local spread before the symptoms develop, the diagnosis is made and treatment is instituted. Indeed, 15% of MDR-TB patients in our study were asymptomatic on presentation.

The financial implications of treating MDR-TB are substantial, with estimates in the region of £60,000 (∼$120,000) per case when assessed in 2000 [20], [21], although £100,000 (∼$200,000) per case may be a more realistic current figure. Since 1997 we have sent 321 samples from AFB positive specimens for *rpoB* testing. Of these, 14 (1 in 23) indicated MDR cases, and one case was rifampicin resistant only. Each test costs £120 (∼$240), making a total expenditure of £38, 520 (∼$77,000). Thus each MDR case cost £2, 750 (∼$5,500) to diagnose an average of 7 weeks earlier than by conventional culture and sensitivity testing. This suggests a potential financial gain from making an early diagnosis, particularly if new secondary contact cases are prevented by earlier treatment of the index case. However, a full cost-benefit analysis would be required to confirm this. Importantly, a recent WHO Expert Group report concluded that there is sufficient evidence to justify the use of line probe assays for the detection of MDR-TB in the developing world, where the majority of MDR-TB cases reside [22]. The technology is advancing rapidly and costs are coming down, with current estimates in the region of USD17-35 per test depending on the test, thus an affordable molecular MDR-TB test for poorer countries is a definite possibility in the near future.

The number of donors subjected to *rpoB* testing in this study are relatively small, and thus it is difficult to draw firm conclusions about the treatment outcomes. Indeed a larger multicentre analysis would be needed to provide this information. Fewer MDR-TB patients diagnosed by *rpoB* required costly surgery, and there were no deaths in the MDR-TB group diagnosed by *rpoB* compared to 11% of those diagnosed by culture. This certainly supports measures directed at early diagnosis and treatment, although the numbers are too small to determine significance. Delayed diagnosis is just one reason why there might have been deaths among the culture diagnosed MDR-TB group only, but there are a number of other plausible explanations.

The *rpoB* test was 100% sensitive and 92% specific for MDR-TB diagnosis in our case series. A study from the Mycobacterium Reference Laboratory in the UK has examined the laboratory impact of the *rpoB* probe and reported a sensitivity and specificity of >95% for the diagnosis of rifampicin resistance in 1,997 clinical specimens [9]. Rifampicin resistance in the presence of isoniazid sensitivity was found in 17.5% of *rpoB* mutant strains [9]. This justifies our practice of continuing isoniazid therapy until full culture and sensitivity results are available. Barnard *et al.* similarly found that the *rpoB* test had a sensitivity of 99% and specificity of 100% for the diagnosis of smear-positive MDR-TB cases in a routine diagnostic laboratory in South Africa, and also that it performed well for smear-negative, culture-positive cases [23].

In summary our findings highlight important considerations in the diagnosis and management of MDR-TB. Previous recognised risk factors were often absent on presentation, but referral from a port of entry into the country posed a distinct risk for MDR-TB, highlighting the need for early screening of these cases. Rapid molecular testing was found to dramatically reduce the time to diagnosis, commencement of appropriate treatment and to improve outcome. Routine introduction of rapid molecular testing for all AFB positive cases has the potential to have a major public health impact on the treatment and prevention of spread of MDR-TB.
